# Case report: Levodopa-responsive parkinsonism with akinetic mutism after ventriculo-peritoneal shunt

**DOI:** 10.3389/fneur.2023.1184713

**Published:** 2023-06-02

**Authors:** Ying Zhang, Ping Li, Jifeng Zhang, Chunyang Li, Peng Sun, Fujun Li, Zhuomin Jiao

**Affiliations:** ^1^Department of Neurology, South China Hospital, Health Science Center, Shenzhen University, Shenzhen, China; ^2^Department of Radiology and Nuclear Medicine, The Second Affiliated Hospital of Harbin Medical University, Harbin, China; ^3^Department of Neurology, The Second Affiliated Hospital of Harbin Medical University, Harbin, China; ^4^Department of General Surgery, South China Hospital, Health Science Center, Shenzhen University, Shenzhen, China

**Keywords:** parkinsonism, ventriculo-peritoneal shunt, ^18^F fluorodeoxyglucose, akinetic mutism, levodopa

## Abstract

**Background:**

Parkinsonism and akinetic mutism (AM) following ventriculo-peritoneal shunt (VPS) without underdrainage used to be considered rare, but may be underdiagnosed in daily clinical practice. Although the pathophysiology is still unclear, in several case reports, the parkinsonism and AM after VPS shows responsiveness to dopaminergic treatment.

**Case presentation:**

We report a 19-year-old male that presented with severe parkinsonism and AM after VPS. Meanwhile, ^18^F-FDG-PET showed a cortical and subcortical hypometabolism. Fortunately, levodopa dramatically improved patient's symptoms and brain hypometabolism. This report provides support for the possibility that dopamine deficiency inhibits brain metabolism, and further elucidates the pathogenesis of parkinsonism and AM.

**Conclusion:**

This report highlights the presentation of a treatable parkinsonism and points out that Levodopa and/or dopamine agonist should be the first choice if the patients develop parkinson-like symptoms after VPS.

## Introduction

Parkinsonism following ventriculo-peritoneal shunt (VPS) has been reported in patients without underdrainage (1–3), but the pathophysiology is unclear. Here, we report a similar case that presented with severe parkinsonism and akinetic mutism (AM) after VPS. Interestingly, Levodopa treatment not only improved the clinical outcomes, but also reversed extensive cortical and subcortical hypometabolism.

## Case report

A 19-year-old male presented with chronic headache, depression, irritability, and inattention. He was diagnosed with obstructive hydrocephalus caused by aqueduct stenosis and underwent VPS. Post-operation, the patient's headache rapidly relieved and other symptoms improved within a month. 3 months after shunt insertion, the patient was hospitalized with complaints of headache, drowsiness, and intermittent confusion. Head CT scan showed dilation of the lateral ventricles and the third ventricle. After recalibrating the VPS setting and reducing the cerebrospinal fluid (CSF) pressure to 120 mmH_2_O, the patient's symptoms improved, and the ventricular size returned to normal within a week.

However, 6 months after VPS, the patient was readmitted due to blunted response and dilation of ventricles. This time, the CSF pressure was reduced to 100 mmH_2_O. Although the enlarged ventricles contracted after a few days, there was no improvement in symptoms. Additionally, progressive aggravation, gradual -hypophonia, -salivation, -eating difficulty, -muscle stiffness and -bradykinesia was observed.

**Figure 1:**
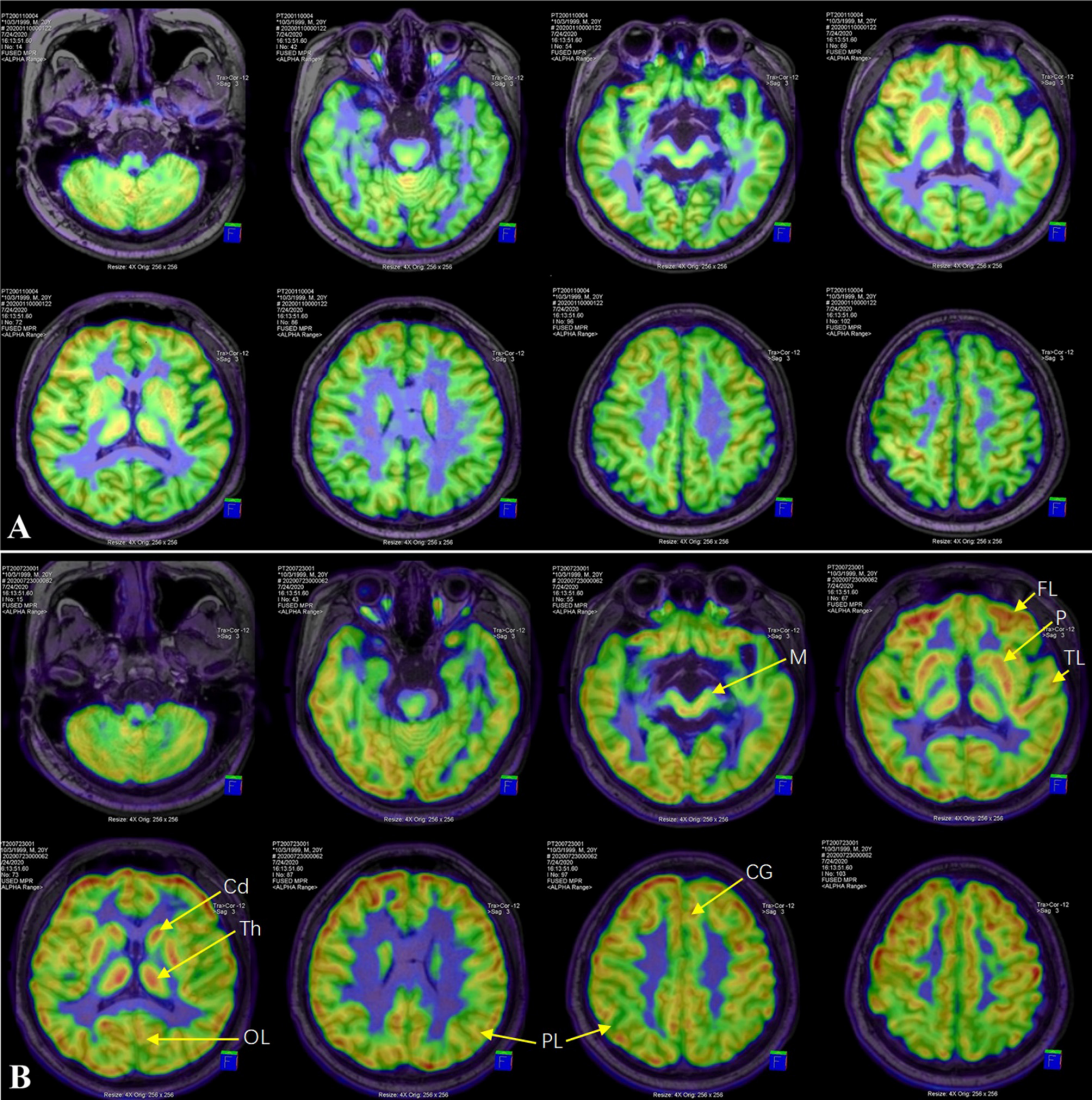
^18^F-FDG PET/MR image. Compared with before levodopa treatment **(A)**, FDG-SUVR of all ROIs, including cortex (frontal lobe, parietal lobe, temporal lobe, occipital lobe and cingulate gyrus) and subcortex (putamen, caudate nucleus, thalamus and midbrain), increased significantly after 6 months of treatment **(B)**. FL, frontal lobe; PL, parietal lobe; TL, temporal lobe; OL, occipital lobe; CG, cingulate gyrus; Cd, caudate nucleus; P, putamen; Th, thalamus; M, midbrain.

Six months later (i.e., 12 months after VPS), the patient was referred to our center with mutism and immobility. On examination, he had eye movement disorders, severe rigidity of limbs and bradykinesia, hyperreflexia, and clonus of both lower limbs with positive Babinski sign. Head CT scan showed no ventricle enlargement. ^18^F fluorodeoxyglucose (^18^F-FDG) PET/MR images showed extensive cortical and subcortical hypometabolic patterns (Figure 1).

Severe Parkinsonism and AM after VPS was diagnosed. The therapy of Madopar (levodopa/benserazide) and Pramipexole was initiated. Over the next 2 weeks, the dose of Madopar and Pramipexole slowly increased to 550/137.5 mg/day and 1.125 mg/day, respectively. Nearly a month after initiation of treatment, the patient let out a long-lost weak cry leading to an emotional moment for the family. After that, he began to pronounce monosyllabic words and was able to stand and walk with assistance. A complete recovery of speech and motor skills was observed 2 months later. The patient could not recall the events he had experienced during the period of severe illness. At a 6-month follow-up post-treatment, head MRI scan showed no ventricular dilation. The regions of interest (ROI) of cortex (frontal lobe, parietal lobe, temporal lobe, occipital lobe and cingulate gyrus) and subcortex (putamen, caudate nucleus, thalamus and midbrain) were delineated on ^18^F-FDG PET/MR images. The uptake value of each ROI was normalized by cerebellum to obtain the standard uptake value ratio of FDG (FDG-SUVR). Compared with before levodopa treatment, FDG-SUVR of all ROIs increased significantly after 6 months of treatment (Table 1). Over the next year, the doses of Madopar and Pramipexole were gradually reduced until they were discontinued. The patient's neurological symptoms have not recurred during the 10-month follow-up.

**Table 1:** FDG-SUVR in the ROIs of cortex and subcortex before and after dopaminergic treatment.

**ROIs**	**FDG-SUVR**_**cerebellum**_		**Improvement**

	**Before**	**After**	**After/Before (%)**
**Frontal lobe**			
Left	1.35	1.59	118.52%
Right	1.37	1.86	135.79%
**Parietal lobe**			
·	1.20	1.58	131.36%
Right	1.27	1.63	127.84%
**Temporal lobe**			
Left	1.13	1.43	126.39%
Right	1.09	1.49	135.83%
**Occipital lobe**			
Left	1.08	1.37	127.28%
Right	1.31	1.83	139.42%
**Cingulate gyrus**			
—	1.24	1.68	135.57%
**Putamen**			
Left	1.67	2.20	131.59%
Right	1.47	2.18	149.07%
**Caudate nucleus**			
Left	1.52	2.19	144.15%
Right	1.77	2.20	124.07%
**Thalamus**			
—	1.49	2.20	148.22%
**Midbrain**			
—	1.35	1.88	140.07%

SUVR, standard uptake value ratio; ROI, regions of interest.

## Discussion

**Figure 2:**
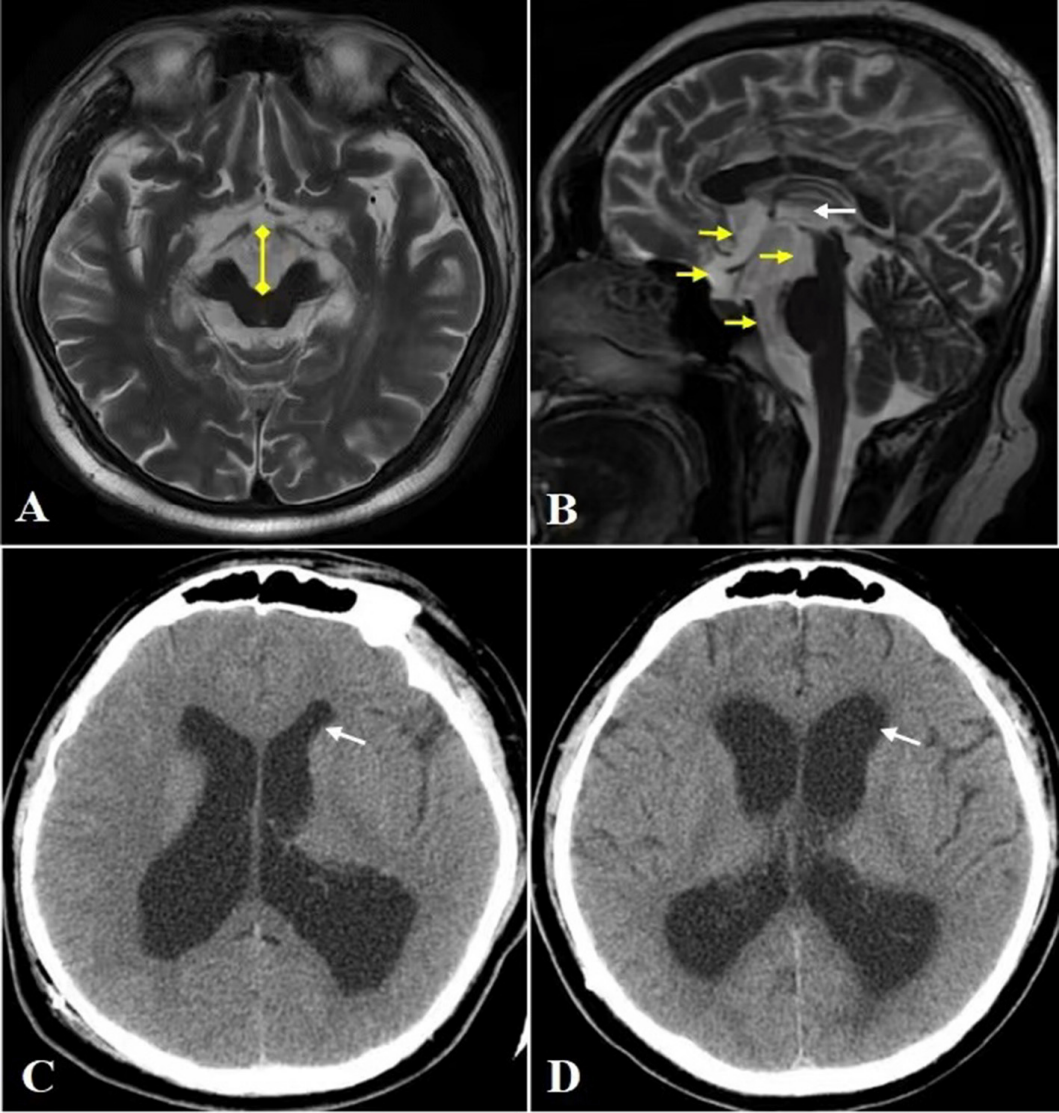
CT and MRI. **(A)** Head MRI axial view reveals significantly widened interpeduncular cistern (yellow arrow). **(B)** Head MRI sagittal view reveals marked enlargement of cistern of lamina terminalis, chiasmatic cistern, interpeduncular cistern and prepontine cistern (yellow arrow), and contraction of the third ventricle (white arrow). **(C, D)** The anterior horn (white arrow) of dilated lateral ventricle before VPS **(C)** and after VPS **(D)**, the latter shows widening of the anterior horn and Evan's index >0.3, which are characteristics of NPH.

Aqueduct stenosis is known to obstruct CSF circulation and block the CSF pressure transmission between the supratentorial ventricular system (lateral ventricle and third ventricle) and the infratentorial ventricular system (fourth ventricle and subarachnoid cavity) (4). When VPS is performed, the supratentorial pressure decreases rapidly, while the infratentorial pressure increases relatively. This change in pressure causes the ventral cisterna, its adjacent structures (directly adjacent to the front and bottom of the third ventricle) to be disturbed by the CSF pressure gradient. This is especially true in the midbrain, even contributing to midbrain displacement. It has previously been reported that local pressure to the ventral midbrain, accompanied by shearing and torsion of nigrostriatal projection fibers, causes the global rostral midbrain dysfunction (3, 5, 6). Enlargement of the ventral cisterns (cistern of lamina terminalis, chiasmatic cistern, interpeduncular cistern, and prepontine cistern) and contraction of the third ventricle are revealed by MRI scans in our patient, post VPS (Figures 2A, B) indicating that there had been a change in CSF pressure gradient here. Clinical evidence supporting the above mechanism is that endoscopic third ventriculostomy (ETV) successfully improved parkinsonism following VPS in some patients (5–7). Unlike VPS, ETV is performed at the bottom of the third ventricle to re-establish the connection between the supratentorial and infratentorial ventricle systems, thereby eliminating the transentorial CSF pressure gradient.

Another possible pathogenesis is that repeated sharp changes in the size of the ventricle led to stretching of the ventricle walls and damage the ascending dopaminergic projection system located in the paraventricular area (8). In addition, with the expansion and contraction of the third ventricle, the CSF pressure fluctuation in the ventral cistern becomes frequent, which further leads to midbrain injury. This makes us have to think about the risk factors of repeated ventricular dilatation as well as how to deal with. Long-term excessive stretching of ventricular wall will result in an irreversible damage to ventricular wall compliance, increasing susceptibility to normal pressure hydrocephalus (NPH) and even low-pressure hydrocephalus (9, 10). In our case, the lateral ventricular dilatation is different before and after VPS, and the latter shows the ventricular dilatation characteristics of NPH (Evan's index >0.3) (Figures 2C, D). Therefore, post VPS, setting a lower pressure range of CSF may be considered for patient safety.

Although the normalization in size of the ventricles, Parkinsonism and AM in the patient had progressively worsened prior to treatment with levodopa. The patient's symptoms improved dramatically after levodopa and pramipexole therapy. Parkinsonism and AM have not recurred during the follow-up of 2 years and 4 months, even 10 months after withdrawal of Levodopa. It is speculated that reversible dysfunction of the presynaptic nigrostriatal dopaminergic pathway caused by the above mechanical factors is the main pathophysiological mechanism in this case, which is also a widely accepted view at present (11–14). According to the pathophysiological mechanism of impaired presynaptic dopaminergic pathway, dopaminergic drugs should play an active therapeutic role. As previously reported in parkinsonism and/or AM after VPS for hydrocephalus, the majority of patients treated with levodopa achieved significant improvement (1–3, 5–7, 10–22). A few patients received dopamine agonist and/or amantadine, and achieved efficacy (15, 18–28). Anticholinergic drugs have also been reported to relieve symptoms (3, 13, 22) (Table 2).

**Table 2:** Summary of clinical features in patients with Parkinsonism and/or akinetic mutism following hydrocephalus treatment.

**Case**	**Author(s)**	**Age (yrs)/sex**	**Clinical syndrome**	**Hydrocephalus (HD)**	**Operation**	**Therapeutic drugs**	
						**Levodopa**	**Others**
1–2	Aidi et al. (27)	36/M, 41/F	Akinetic mutism	Obstructive hd due to AS	Multiple shunt revisions	—	Bromocriptine
3	Anderson (24)	20/M	Akinetic mutism	Obstructive hd due to AS	Multiple shunt revisions	—	Bromocriptine, Phedrine
4	Lin et al. (25)	3/M	Akinetic mutism	Obstructive hd due to AS	Multiple shunt revisions	—	Bromocriptine
5	Berger et al. (13)	21/F	Akinetic mutism and Parkinsonism	Obstructive HD due to AS	Multiple shunt revisions	Prolopa	Cogentin
6	Costa et al. (20)	38/M	Parkinsonism	Obstructive HD due to AS	VPS	Levodopa/ benserazide	Bromocriptine
7–10	Curran et al. (11)	7/M, 16/M	Parkinsonism	Obstructive HD due to AS	Multiple shunt revisions	Sinemet	—
		21/M	Parkinsonism	Obstructive HD secondary to pineal mass	VPS	Sinemet	—
		72/M	Parkinsonism	Normal pressure HD	VPS	Sinemet	—
11	Hashizume et al. (7)	47/F	Parkinsonism	Obstructive HD due to AS	VPS and ETV	Levodopa/ carbidopa	—
12	Kim et al. (1)	46/M	Parkinsonism	Obstructive HD due to AS	Multiple shunt revisions	Levodopa/ carbidopa	—
13	Kinugawa et al. (3)	49M	Parkinsonism	Obstructive HD due to AS	VPS	Levodopa	Trihexyphenidyl
14	Moser et al. (26)	21/M	Akinetic mutism	Obstructive hd due to AS	Multiple shunt revisions	—	Bromocriptine, Metoprolol
15, 16	Ochiai et al. (15)	59/M, 32/M	Parkinsonism	Obstructive HD	Multiple shunt revisions	Levodopa	Bromocriptine, Amantadine
17	Okawa et al. (5)	51/M	Parkinsonism	Obstructive HD due to AS after the bleeding in the fourth ventricle surgery	VPS and ETV	Levodopa/ benserazide	—
18	Prashantha et al. (2)	38/M	Parkinsonism	Obstructive HD due to AS	Multiple shunt revisions	Levodopa/ carbidopa	—
19	Psarros et al. (28)	26/F	Akinetic mutism	Bstructive hd due to cystic mass	Endoscopic exploration, cystresection, septostomy	—	Bromocriptine
20	Racette et al. (12)	44/M	Parkinsonism	Obstructive HD due to AS	Multiple shunt revisions	Levodopa/ carbidopa	—
21	Rebai et al. (23)	12/F	Akinetic mutism and Parkinsonism	AS due totectal tumor	ETV failure and multiple shunt revisions	Levodopa	Bromocriptine
22	Sakurai et al. (16)	46/F	Parkinsonism	Obstructive HD due to AS	VPS	Levodopa	—
23	Shahar et al. (10)	17/M	Parkinsonism	Obstructive HD due to AS	Multiple shunt revisions	Sinemet	—
24	Shpiner et al. (6)	35/M	Parkinsonism	Obstructive HD due to AS	VPS and multiple recalibrations, ETV	Levodopa/ carbidopa	—
25	Shpiner et al. (6)	26/M	Parkinsonism	Obstructive HD due to pineal tumor	Multiple shunt revisions	Levodopa/ carbidopa	—
26, 27	Villamil et al. (21)	42/F	Parkinsonism	Obstructive HD due to AS	VPS and ETV	Levodopa/ carbidopa	—
		32/F	Parkinsonism	Obstructive HD due to rosette-forming glioneuronal tumor	VPS and multiple recalibrations, ETV	Levodopa/ carbidopa	—
28	Villamil et al. (21)	20/M	Parkinsonism	Obstructive HD due to AS	Multiple shunt revisions	—	Cabergoline, Amantadine
29	Watahiki et al. (22)	39/M	Akinetic mutism and Parkinsonism	Obstructive HD due to AS	Multiple shunt revisions	Levodopa	Bromocriptine, Trihexyphenidyl
30	Yomo et al. (17)	64/M	Parkinsonism	Obstructive HD due to AS	Multiple shunt revisions	Levodopa/ carbidopa	—
31, 32	Zeidler et al. (18)	57/M	Parkinsonism	Obstructive HD due to AS	VPS and Torkildsen shunt	Sinemet	Bromocriptine
		21/F	Parkinsonism	Obstructive HD due to AS	Multiple shunt revisions	Madopar	—
33	Zhang et al. (14)	44/M	Parkinsonism	Obstructive HD due to AS	VPS and antipsychotic drugs	Madopar	—
34	Zhou et al. (19)	45/M	Parkinsonism	obstructive HD due to AS	VPS	Madopar	Pramipexole
35	Our case	19/M	Akinetic mutism and Parkinsonism	Obstructive HD due to AS	VPS and multiple recalibrations	Madopar	Pramipexole

Hydrocephalus, HD; ventriculo-peritoneal shunt, VPS; aqueduct stenosis, AS; endoscopic third ventriculostomy, ETV.

Our patient was diagnosed with parkinsonism and AM after VPS and completely relieved after receiving Madopar and Pramipexole treatment, which is similar to most of the cases in Table 2. Notably, unlike previous cases, this report provides support for the possibility that dopamine deficiency inhibits brain glucose metabolism. Before dopaminergic treatment, the patient's brain functions such as sensation, movement, memory, and language were impaired. ^18^F-FDG PET also recorded extensive cortical and subcortical hypometabolic patterns (Figure 1A). Our treatment constituted of only dopamine supplementation resulting in relief of clinical symptoms and reversal of brain hypometabolism patterns (Figure 1B). Previously, it was reported that ETV improved the symptoms of severe parkinsonism after VPS and restored cerebral cortical flow (7). As mentioned above, ETV can eliminate the damage of CSF pressure gradient to midbrain and contribute to the recovery of nigrostriatal dopaminergic function. Therefore, it seems reasonable to believe that ETV may also function through the dopaminergic pathway. Parkinson's disease is the most representative disease of central dopamine deficiency. The Parkinson's disease related pattern (PDRP) has been validated in multiple independent populations worldwide (29, 30). PDRP is characterized by relatively increased metabolism in the thalamus, putamen/pallidum, pons, cerebellum, and motor cortex and relative decreases in the lateral frontal and parietooccipital areas. However, unlike PDRP, our case shows a broad (whether cortical or subcortical), symmetrical pattern of brain hypometabolism, which is significantly improved after dopaminergic treatment.

In this report, two limitations should be mentioned. Firstly, due to technical limitations, the patient failed to complete the evaluation of presynaptic nigrostriatal dopaminergic function, such as DAT, VMAT_2_ or ^18^ F-DOPA PET tests. Secondly, the limitations of the case report itself, that is to say, it is difficult to draw a firm conclusion from only one clinical case, and more accumulation is needed to confirm our findings.

In conclusion, we reported a case of severe parkinsonism and AM with cerebral hypometabolism after VPS, which was dramatically improved by levodopa. The hint for clinical practice is that Levodopa and/or dopamine agonist should be the first choice if the patients develop Parkinson-like symptoms after shunt.

## Data availability statement

The original contributions presented in the study are included in the article/supplementary material, further inquiries can be directed to the corresponding authors.

## Ethics statement

Ethical review and approval was not required for the study on human participants in accordance with the local legislation and institutional requirements. The patients/participants provided their written informed consent to participate in this study. Written informed consent was obtained from the individual(s) for the publication of any potentially identifiable images or data included in this article.

## Author contributions

YZ drafted the manuscript and was responsible for patient diagnosis and treatment. PL and JZ performed the PET CT detection and analysis. CL and PS participated in the collection and collation of clinical data. FL and ZJ critically revised the manuscript. All authors contributed to the article and approved the submitted version.
